# Effectiveness of a Patient-Centered Assessment With a Solution-Focused Approach (DIALOG-A) in the Routine Care of Colombian Adolescents With Depression and Anxiety: Protocol for a Multicenter Cluster Randomized Controlled Trial

**DOI:** 10.2196/43401

**Published:** 2023-02-08

**Authors:** Carlos Gómez-Restrepo, Jose Alejandro Rumbo Romero, Martha Rodriguez, Laura Ospina-Pinillos, Diliniya Stanislaus Sureshkumar, Stefan Priebe, Victoria Bird

**Affiliations:** 1 Department of Clinical Epidemiology and Biostatistics Department of Psychiatry and Mental Health Pontificia Universidad Javeriana Bogota Colombia; 2 Unit for Social and Community Psychiatry Wolfson Institute of Population Health Queen Mary University of London London United Kingdom

**Keywords:** randomised controlled trial, adolescent, mental health, depression, anxiety, telemedicine, primary health care, Colombia

## Abstract

**Background:**

Colombia is a middle-income country in South America, which has historically had high rates of mental health problems, coupled with a scarcity of mental health care. There is growing concern for the mental health of the adolescent population within this region. There is a significant treatment gap for young people, especially those living in the most vulnerable areas. DIALOG+ is a low-cost patient-centered intervention that can potentially improve the delivery of care and quality of life for adolescents with mental health problems.

**Objective:**

This exploratory randomized controlled trial aims to evaluate the effectiveness, acceptability, and feasibility of an adapted version of the DIALOG+ intervention (DIALOG-A) in the community treatment of Colombian adolescents with depression and anxiety.

**Methods:**

In total, 18 clinicians and 108 adolescents will be recruited from primary health care services in Bogota and Duitama, Colombia. Clinicians will be randomized 2:1 to either the intervention (12 clinicians:72 adolescents) or control group (6 clinicians:36 adolescents). In the intervention arm, clinicians will use DIALOG-A with adolescents once per month over 6 months. The control arm will continue to receive routine care. Outcomes will be measured at baseline, 6 months, and 9 months following randomization. Semistructured interviews with all clinicians and a subset of adolescents in the intervention arm will be conducted at the end of the intervention period. Quantitative and qualitative analysis of the data will be conducted.

**Results:**

Trial recruitment was completed toward the end of October 2022, and follow-up is anticipated to last through to October 2023.

**Conclusions:**

This is the first study to test an adapted resource-orientated intervention (DIALOG-A) in the treatment of adolescents with depression and anxiety attending primary care services. If the results are positive, DIALOG-A can be implemented in the routine care of adolescents with these mental health problems and provide valuable insight to other middle-income countries.

**Trial Registration:**

ISRCTN Registry ISRCTN13980767; https://www.isrctn.com/ISRCTN13980767?q=ISRCTN13980767

**International Registered Report Identifier (IRRID):**

DERR1-10.2196/43401

## Introduction

### Background

Currently, an estimated 5.2 million adolescents are living in Colombia. During adolescence, physical, emotional, and social changes make individuals more vulnerable to mental health problems. Worldwide, and in Columbia, adolescence also marks the beginning of relationships and the emergence of sexuality, with a mature body capable of exercising it [1,2]. All these circumstances are intensified when adolescents live in an area with a history of conflict, displacement, inequality, poverty, violence, and lack of opportunities.

The internal conflict and violence experienced in Colombia for more than 50 years has led to the forcible displacement of 8.2 million individuals since 1985 [3], and a high proportion of the population is living in extreme poverty, which is currently estimated to be 12.2% (per the National Administrative Department of Statistics) [4]. This has contributed to fragile neighborhoods, particularly within urbanized inner-city areas, and deprived rural communities [3,4]. This complex picture has led to an increase in mental health illness among adolescents, including a rise in the prevalence of depression and anxiety, with a 1-year estimate of 4%-5% [5,6].

In Colombia, anxiety disorders are the most frequent, with an estimated prevalence of 15%-20%, and with rates reported to be as high as 31.9% among individuals aged between 13 and 18 years [7]. Similarly, rates of anxiety and depression are also commonly high among people aged 12 to 17 years. Using the Self-Reporting Questionnaire to assess this population, 13% of adolescents screened positive for depression [8,9].

Despite the high prevalence rates and long-term impacts of depression and anxiety, only half of the adolescents diagnosed with anxiety and depression in low- and middle-income countries (LMICs) access treatment, with a smaller percentage (20.5%) receiving at least the minimum standard of care. Consequently, less than 12% of adolescents in LMICs such as Colombia receive even minimally adequate treatment, pointing to an issue of both access and quality [10,11]. In Colombia, one of the most relevant barriers to mental health services delivery is a lack of sufficiently trained professionals [12,13]. Where resources are available, they tend to be distributed unevenly, concentrated in large cities, and fail to reach low-income or rural areas. Hence, low-cost, generic interventions that can be delivered by a range of staff (including those beyond the mental health system) are urgently needed to tackle depression and anxiety and ultimately improve adolescent health [14-16].

DIALOG+ is a technology-assisted and resource-orientated intervention based on quality-of-life research, patient-centered communication, and solution-focused therapy. Within DIALOG+, patients use a tablet to rate their satisfaction with 8 life domains (*mental health*, *physical health*, *job situation*, *accommodation*, *leisure activities*, *friendships*, *relationship with family/partner*, and *personal safety*) and 3 treatment aspects (*medication*, *practical help*, and *meetings with professionals*). This is followed by a 4-step solution-focused approach to identify and harness the existing resources available to the patient to address these concerns and improve their quality of life. This tool was established as an effective intervention for adults with mental health problems following a sustained program of research including multiple clinical trials [17-19].

Within a cluster randomized controlled trial (RCT) conducted across 6 European countries, regular use of the initial form of DIALOG+ over a year led to a better quality of life, fewer unmet needs, and higher treatment satisfaction for patients with psychosis. Following further modifications, DIALOG+, delivered once per month over 6 months, was compared to an active control in a pragmatic RCT involving 49 clinicians and 178 patients diagnosed with psychosis. Patients in the experimental group had a significant improvement in quality of life and lower general psychopathological symptoms after 3, 6, and 12 months. They also had fewer unmet needs and a better objective social situation. The effect size for improvements in quality of life ranged from 0.30 (95% CI 0.02-0.58) at 3 months to 0.32 (95% CI 0.06-0.58) at 12 months. Furthermore, DIALOG+ saved the National Health Service £1345 (US $1610.16) per patient, mainly due to fewer inpatient days required by the experimental group [17-19]. However, research has not assessed whether the intervention may also be effective for adolescents with common mental disorders.

### Study Aims and Objectives

This planned exploratory cluster RCT sits within the context of a larger research program called “Building resilience in adolescence - improving quality of life for adolescents with mental health problems in Colombia (BRiCs).” This is a collaborative project between Queen Mary University of London (London, the United Kingdom) and the Pontificia Universidad Javeriana of Bogota (Bogota, Colombia), which aims to improve health outcomes and quality of life for the adolescent population with depression and anxiety in Colombia. During the initial phase of the project, semistructured interviews and focus groups were conducted alongside a pilot study to adapt the existing intervention (DIALOG+) for use with adolescents [20]. The new intervention is called DIALOG-A.

Given this background, the overall aim of this study is to evaluate the effectiveness of DIALOG-A in the community treatment of adolescents with anxiety and depression in Colombia.

## Methods

### Study Design and Setting

A multisite, exploratory cluster RCT will be conducted to assess the effectiveness, acceptability, and feasibility of DIALOG-A for adolescents with anxiety or depression. In total, 18 clinicians and 108 adolescents will be recruited from primary health care services in Colombia and outcomes measured at baseline, 6 months, and 9 months following randomization.

The 3 sites included in this RCT in are based in 2 cities: Bogota and Duitama. Bogota is the capital city of Colombia, with 7.9 million inhabitants. Duitama, in contrast, is a small city of approximately 128,400 inhabitants in a rural location. Community mental health care is provided by primary health centers, which include an adolescent-friendly service. The population within Duitama is traditionally rural where poverty, alcohol intake, adult illiteracy, and family violence are common. Almost all the adolescents that use health services on this site belong to low socioeconomic status [21,22].

### Participants

#### Inclusion Criteria

The inclusion criteria for clinicians will be as follows: (1) being aged 18 years or older, (2) having a medical qualification as a general practitioner or pediatrician, (3) having regular contact with adolescent patients with anxiety and depression, (4) having at least 3 months of experience in working with adolescents, and (5) having no plans to leave their current position within the next 6 months.

The inclusion criteria for adolescents will be as follows: (1) being aged 13 to 16 years; (2) currently experiencing anxiety and depression, which is defined as a cutoff score of ≥7 on the Self-Reporting Questionnaire [23]; and (3) having the capacity to provide informed assent.

#### Exclusion Criteria

The exclusion criteria for adolescents will be as follows: (1) having severe mental illness (psychosis, bipolar disorder, or schizophrenia), (2) having cognitive impairment or severe learning disability, and (3) being unable to speak and understand Spanish.

#### Recruitment and Informed Consent

Each study site will include a coordinator who will recruit the clinicians for the study. A research assistant will recruit the adolescents, guide all participants through the informed consent process, and assess the outcomes at 3 defined time points (baseline, 6 months, and 9 months post randomization). A wider administrative staff member will schedule the follow-ups and provide the clinicians’ caseload to the research assistant to identify potential adolescent participants.

#### Clinicians

Each clinical site was identified from the national network of public and private primary care centers that provide care to the adolescent population of Colombia. An in-person meeting was arranged between the potential clinicians and study coordinators of each study site.

Eligible clinicians will provide their informed consent by signing and dating an electronic informed consent form via the Research Electronic Data Capture (REDCap) data management platform.

#### Adolescents

With the support of the administrative staff, research assistants will check each physician’s caseload to identify adolescent patients who meet the inclusion criteria. Potentially eligible adolescents will be then invited to a one-on-one meeting with the local site’s research assistant to discuss the study in more detail and answer any questions. This meeting will also be used to verify whether the inclusion criteria are met and to obtain informed assent and consent from the adolescents and their parents or guardians, respectively. During the study period, new referrals to the clinics will be screened for eligibility, and potentially eligible adolescents will be contacted by a research assistant.

Both the research team and the participants will keep a copy of the signed informed consent or assent form via REDCap. The researcher’s contact details will be provided on the informed consent form to allow participants to raise any questions or concerns they may have throughout the study. This process will take place before any data collection begins.

### Intervention

The enrolled clinicians will be contacted and invited to a web-based meeting on a free videoconferencing platform such as Zoom or Microsoft Teams with a researcher who will re-explain the objectives of the RCT and answer any questions or concerns that are raised.

Clinicians randomized to the intervention arm will use DIALOG-A with the adolescents once per month for 6 months. Clinicians allocated in this arm will receive a tablet, with the DIALOG-A app already preloaded. Each DIALOG-A session will last approximately 20 minutes and will take place face to face with the clinician. Where it is not possible for a face-to-face consultation to occur, a web-based consultation will be conducted in line with standard care procedures within the health care service. In each session, the clinician will ask the patient to rate all the domains on the DIALOG scale, which will then be followed by the 4-step solution-focused approach. After completing the 6-month intervention with the DIALOG-A app, clinicians and adolescents can continue using the intervention depending on their needs, and this will be documented and considered in the analysis.

### Treatment-as-Usual Control

The intervention group will be compared to treatment-as-usual control. Clinicians in the control arm will follow the routine care provided for adolescents accessing primary care services. The frequency of the consultations during the duration of the RCT will vary depending on the clinical criteria of the respective clinician and the adolescent; however, this will be, on average, once per month.

### Training and Supervision

All clinicians in the intervention arm will receive training in DIALOG-A through one-to-one web-based sessions with a researcher, which will take between 45 and 60 minutes. During these training sessions, clinicians are provided with reading material, instructions, and examples on how to use the DIALOG-A app with adolescents [24]. Clinicians can contact any member of the local research team for further sessions and for advice or support at any time of the RCT. Refresher training will be provided to clinicians in the intervention arm depending on their needs, with a minimum of 2 refresher sessions provided throughout the RCT.

All clinicians allocated to either the intervention or control arms will receive training in the diagnosis and treatment of adolescent depression and anxiety through an asynchronous web-based course. These training sessions include academic passages on anxiety disorders, mental health disorders related to trauma and stress, and mood disorders, which are developed by adolescent mental health specialists from Pontificia Universidad Javeriana. Clinical cases and discussion forums will be available as well. A final quiz with a minimum passing score of 80% must be completed by participating clinicians.

### Randomization

To reduce the potential for contamination, the unit for randomization will be the clinician. Clinicians will be randomized 2:1 to either the intervention (12 clinicians:72 adolescents; to have DIALOG-A sessions once per month for 6 months) or to the treatment-as-usual control group (6 clinicians:36 adolescents; appointments scheduled as and when needed, without DIALOG-A or specific assessments). For existing patients, who are already on the caseload, adolescent participants will be recruited and baseline assessments conducted prior to their clinician being randomized. Individuals who are new to the service and are not on the existing caseload of a clinician will be randomly assigned to either the intervention or control arm and then to an appropriate clinician, following completion of the baseline measures. We will record the number of adolescent participants who are recruited and individually randomized.

Clinicians and adolescents will not be blinded to allocation due to the nature of the intervention. However, to reduce bias, randomization will be carried out by a blinded independent statistician at the Unit for Social and Community Psychiatry, Wolfson Institute of Population Health, Queen Mary University of London. Baseline data collection will occur before randomization, and all research assistants involved in outcome data collection will be blinded to allocation. We will additionally record the number of postrandomization recruits; for example, individuals who were recruited after their clinician has been randomized.

### Proposed Sample Size

A formal sample size calculation was not conducted as the primary objective is to collect data required for a full trial protocol, including the information needed for sample size calculation. Estimates for adequate sample sizes for exploratory trials range from 24 to 50 per arm, with at least 30 individuals required to estimate the SD of the outcomes. This exploratory trial will include 108 patients to provide usable estimates from each site. Unbalanced 2:1 randomization favoring the intervention will ensure sufficient data about the experience with the DIALOG-A intervention. Randomization will be stratified by the site to include 12 clinicians at the 3 study sites in Bogota and 6 at the study site in Duitama to reflect differences in population size and available resources.

### Quantitative Data

All participants (clinicians and adolescents) will be asked to complete a sociodemographic questionnaire at baseline. Clinicians will be asked to rate the Clinical Global Impression questionnaire after every appointment.

The primary outcomes will be collected at 6 months, with a further follow up of 9 months post randomization—these include symptoms of depression measured using the 8-item Patient Health Questionnaire [25] and symptoms of anxiety measured using the 7-item Generalized Anxiety Disorder scale [26].

All secondary outcomes will be collected at baseline, 6 months, and 9 months (unless otherwise stated)—these include quality of life measured using the Manchester Short Assessment of Quality of Life [27], mental health symptom levels measured using YP-CORE [28], social support measured using the Multidimensional Scale of Perceived Social Support [29], empowerment measured using the Youth Efficacy/Empowerment scale [30], self-esteem measured using the Rosenberg Self-Esteem Scale [31], and economic outcomes measured using a modified Client Service Receipt Inventory [32].

In addition to the outcome data described above, we will collect process measures, including the number of DIALOG-A interventions performed, the date and duration of those sessions, the scores obtained on the DIALOG scale within each session, key topics discussed during the 4-step approach, and what actions were agreed upon. The DIALOG-A app on the tablet is able to capture the data required. This information will be synchronized to a server that the research team can access.

### Qualitative Data

A subset of adolescent participants (n=20) and all clinicians (n=12) randomized to the intervention arm will be invited to complete an in-depth qualitative interview after the 6-month assessments (time point at the end of the intervention period) to capture and discuss their experience of delivering or receiving DIALOG-A. Interviews will focus on the barriers and facilitators of attending intervention sessions, suggested adaptations, trial procedures, practical implementation in the study sites, and potential processes of change. These interviews will be audio-recorded, transcribed verbatim, and further analyzed.

### Statistical Analysis

The analysis of data will be discussed and agreed upon with the local and international research teams, with a statistical analysis plan developed by the statistician and signed off by trial steering committee. The local research team will take a leading role in the management and analysis of the data. Data analysis will not be conducted prior to signing off and blinding of the members of the research team, including the statistician responsible for the analysis, who will remain blinded throughout.

### Quantitative Data Analysis

The number of approached and screened participants, those who refused participation, and those who could not be contacted will be recorded. The analysis will assess the number of intervention sessions received by participants and will collect data on dropouts (including reasons for dropping out, if available) from treatment, which will inform the assessment of the acceptability of the DIALOG-A intervention.

Descriptive statistics will be reported for sociodemographic data for all participants. Nominal and ordinal data will be presented as proportions (frequencies), whereas continuous variables will be described using measures of central tendency and those of variability. The latter will be selected in accordance with observed data distributions (parametric or nonparametric), which will be evaluated through normality tests and histograms (probability functions). To assess the effectiveness of the intervention, means and SDs (or median and IQR values) over the 3 time points (baseline, 6 months, and 9 months) will be calculated, and analysis will test the significance of the differences between the means (or other parameters) of outcomes measured. The primary outcome will be the scores on the 8-item Patient Health Questionnaire and the 7-item Generalized Anxiety Disorder scale at 6 months. Mixed effects multivariable logistic regression analysis will be conducted to assess the effect of the intervention on the primary outcome, while controlling for clustering. A full analysis plan will be developed prior to data analysis, which will consider which covariates should be adjusted for in the model, along with methods for dealing with missing data. Quantitative data analysis will be conducted using Stata (version 17; StataCorp).

### Qualitative Data Analysis

Qualitative data will be analyzed using thematic analysis following the guidance of Miles and Huberman and will be conducted using NVivo qualitative analysis software [33]. All interviews will be audio-recorded and transcribed verbatim. A researcher will remove all identifying information from the transcripts, including any references to patients, clinicians, or local services.

An inductive approach will be used to provide new insights and a richer understanding of the data without using preconceived categories. Two members of the research team will first familiarize themselves with the transcripts. Open coding will be used (making notes and headings in the text to describe the content). Similar codes will be grouped under themes, and the identified themes and subthemes will then be checked and refined. Interrater reliability in applying second-level codes (or categories) will be calculated for 20% of the data.

### Reporting of Adverse Events

For this RCT, we will adopt the World Health Organization’s definition for an adverse event: “Any untoward medical occurrence in a patient or clinical investigation subject administered a pharmaceutical product and which does not necessarily have to have a causal relationship with this treatment” [34]. Adverse events and the need for urgent safety measures are not anticipated.

#### Adverse Events

Any adverse events will be recorded in the main research file and in the participant’s clinical records, if appropriate, and the participant will be followed up by the research team. We will establish an adverse event registration form based on REDCap in which research assistants can make a report of any adverse event for the RCT.

#### Serious Adverse Events

Serious adverse events are those that are “unexpected” and will be reported in accordance with the regulations of the research ethics committee (REC) and other relevant regulations in Colombia.

#### Urgent Safety Measures

In the case of urgent safety measures being required, the local principal investigator (PI) will inform the UK PI and the REC of the event per the REC and other relevant requirements and guidelines.

#### Quarterly Safety Reporting

The local PI will send over quarterly reports as required by the REC, using their existing templates and guidelines. These will be additionally monitored by the international trial steering committee, who will assume the role of a data monitoring and ethics committee.

#### Overview of the Safety Reporting Responsibilities

The local PI will ensure that safety monitoring and reporting are conducted in accordance with the requirements of the REC and any other relevant organizations or institutions that are involved in overseeing and monitoring research activities in Colombia.

### Safety and Suicide Risk Reduction

Participants who decline to participate but may have severe depression, severe anxiety, or are at a risk of suicide will be treated in accordance with each clinical site’s procedures. For participants enrolled in the study who have been identified as having severe depression, severe anxiety, or being at a risk of suicide, the primary care physician will review and monitor the results of the screening. The participant will be managed in accordance with the primary care site’s usual protocols and guidelines.

**Figure 1:**
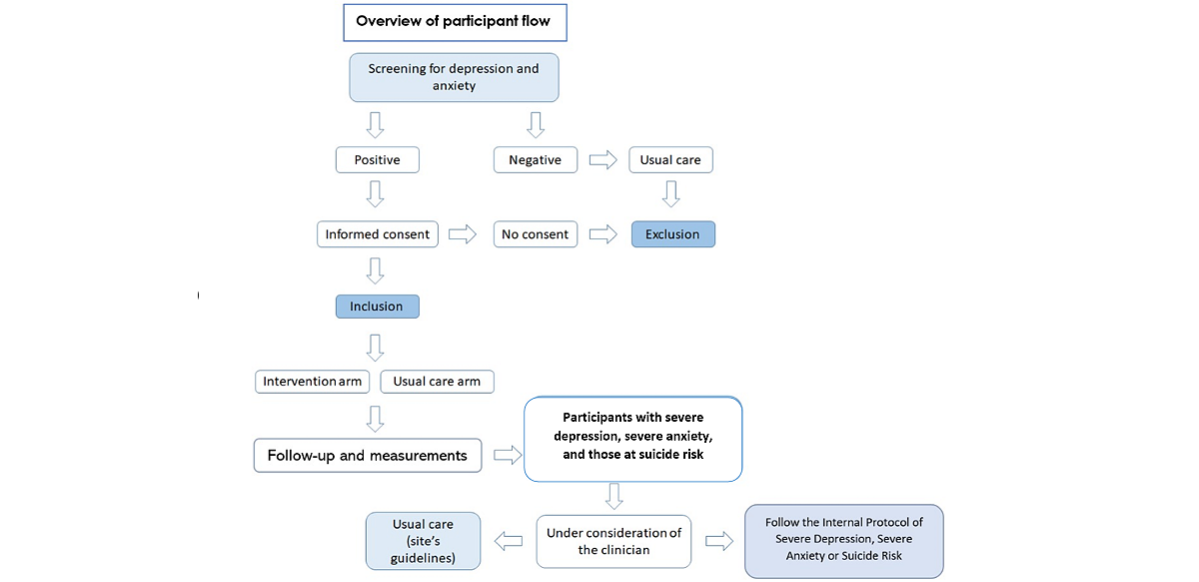
Withdrawal from the study.

Additionally, if the participant meets the criteria for severe depression or anxiety, or is identified as being at risk of suicide, treatment may be either administered by or coordinated with a psychiatrist (in accordance with the Colombian clinical guidelines of best practices). According to the primary care physician, an optional case review could be performed by the academic psychiatrists involved in this study. Figure 1 summarizes the participant flow chart during the RCT.

### Withdrawing From the Study

During the consent process, researchers will ensure that participants are aware of their right to decline participation at any stage of the research and that withdrawing participation will not affect their treatment or rights. Participants who withdraw will be able to ask that their data be destroyed and removed from the database if their withdrawal occurs before the start of data analysis.

If participants withdraw from the study, the research team will ask permission to contact them to take part in the postintervention qualitative interview to capture valuable information regarding reasons for withdrawal, which might be associated with the intervention. This will be explained to participants during the consent process, and participants’ decisions to not be contacted for the postintervention interview will be respected. Participants who wish to withdraw from the intervention will also be asked if they wish to attend the follow-up research assessments, and their decisions to not participate will be respected.

If a participant wishes to withdraw from the study, researchers will record the date of withdrawal and reasons for withdrawal (if provided).

### Ethics Approval

This study received approval from the local REC at Pontificia Universidad Javeriana in Bogota (approval number FM-CIE-0084-20). Consent forms from the parents or guardians and clinicians and assent forms from the adolescents will be obtained prior to the start of the study. Any changes to the protocol will be communicated to and approved by the local institutional review board and patients as appropriate.

### Dissemination

The aims and impact of the program will be achieved through a comprehensive communication plan, which will inform the different stakeholders of the research findings as well as provide information relating to each of the program outputs. This will ensure that the new knowledge obtained translates into improved health outcomes. Target audiences include (but are not restricted to) adolescents and young people, policy makers, service managers, nongovernmental organizations, education, health and youth organizations, charities, and the public. To promote broader capacity building and initiate wider collaborations, we aim to disseminate findings across Colombia and the wider network with which Javeriana University is linked, the Latin American Clinical Epidemiology Network (LatinClen); and an additional network that Javeriana University is linked with, which trains young researchers in community mental health care across South America (RedMaristan).

### International Trial Steering committee

An international trial steering committee has been set up, and the final protocol was approved by this committee. Lead investigators (CG and VB) are nonindependent members, alongside 6 independent members from the United Kingdom and Colombia. CG is the principal investigator in Colombia and VB is the principal investigator in the United Kingdom. The trial steering committee will periodically review the progress of the study and have agreed to take on the role of the data monitoring and ethics committee.

## Results

Trial recruitment was completed toward the end of October 2022, and follow-up is expected to last through October 2023. We anticipate the outcomes of the trial will be reported by mid-2023 and the follow-ups by early 2024. The primary outcomes will be changes in depression [25] and anxiety [26] symptoms and the secondary outcomes will include changes in quality of life [27], mental health symptom levels [28], perceived social support [29], empowerment [30], self-esteem [31], and economic outcomes [32]. Furthermore, we will report on dropout rates and adverse events which take place during the trial.

## Discussion

### Background

Depression and anxiety are leading causes of youth disability worldwide. This exploratory RCT will aim to repurpose an effective, low-cost intervention for the treatment of depression and anxiety in adolescence (DIALOG-A). The research focuses on Colombia where adolescents experience multiple stress factors such as internal displacement, violence, conflict, and deprivation. Improving the health outcomes of adolescents not only is a priority in its own right but also can aid the transferability of the intervention to other LMICs, where adolescents experience similar situations.

### Strengths and Limitations

This study is the first to test the effect of DIALOG-A as a tool for use in primary care services for adolescents with depression and anxiety. One strength is the pragmatic design, which will enable us to adapt to the different logistics in the centers included in this RCT. One limitation, however, is that due to the characteristics of the intervention, blinding of the adolescent participants and clinicians will not be possible, which may result in the introduction of bias and a placebo effect. Additionally, the study is an exploratory study with unequal randomization (2:1 favoring the intervention). Although this will enable us to estimate potential interventions effects, a definitive trial will be needed to establish efficacy.

A potential benefit is that overall, the study has the potential to adapt a low-cost resource-orientated intervention for use in a vulnerable adolescent population. Additionally, for adolescents who will be involved in the testing of the modified intervention (DIALOG-A), this might lead to improved quality of life, social functioning, and symptoms. The RCT will also benefit the clinicians involved as they will be provided with training and supervision to enable them to implement the intervention. If results are successful, DIALOG-A can be implemented in the routine care of these adolescents in Colombia as it does not need the setting of new services or restructuring of preexisting organizations and is of low cost.

## Appendix

Multimedia Appendix 1 Peer review report 1 by Medical Research Council (Swindon, United Kingdom).

Multimedia Appendix 2 Peer review report 2 by Medical Research Council (Swindon, United Kingdom).

Multimedia Appendix 3 Peer review report 3 by Medical Research Council (Swindon, United Kingdom).

## Abbreviations

LMIC: low- and middle-income country
PI: principal investigator
RCT: randomized controlled trial
REC: research ethics committee
REDCap: Research Electronic Data Capture

## Data Availability

Data will be available upon reasonable request. The approved consent forms did not specify that the data would be deposited in a public repository; hence, we will be unable to deposit the data. The deidentified participant data set will be made available from the corresponding author (VB, v.j.bird@qmul.ac.uk) upon reasonable request and subject to a data sharing agreement.
