# Supplementary material for: Effectiveness of a Patient-Centered Assessment With a Solution-Focused Approach (DIALOG-A) in the Routine Care of Colombian Adolescents With Depression and Anxiety: Protocol for a Multicenter Cluster Randomized Controlled Trial
Source: JMIR Res Protoc. 2023 Feb 8;12:e43401. doi: 10.2196/43401 (PMC9947748; doi:10.2196/43401)
Supplement: Multimedia Appendix 1 [file resprot_v12i1e43401_app1.pdf]

Medical Research Council
2nd Floor David Phillips Building, Polaris House, North Star Avenue, Swindon,
United Kingdom SN2 1ET
Telephone +44 (0) 1793 416200
Web http://www.mrc.ac.uk/

COMPLIANCE WITH THE DATA PROTECTION ACT 1998
In accordance with the Data Protection Act 1998, the personal data provided on this form will be processed by MRC, and may be held on computerised database and/or manual files. Further details may be found in the **guidance notes**

Research Grant Peer Review

MRC Reference: MR/S023674/1

Document Status: With Council

MRC/DFID/NIHR Adolescent Health 2018

Applicant Details

Applicant	Dr Victoria Jane Bird	Organisation	Queen Mary University of London

Title of Research Project

Building resilience in adolescence - improving quality of life for adolescents with mental health problems in Colombia (BRiCs study)

Review Information

Response Due Date	21/12/2018	Reviewer Reference:	057151822

Research Quality

Research Quality: Please comment on the importance and competitiveness of the proposed research, including:

(1) strength of medical or scientific case (2) level of innovation, and whether this is likely to lead to significant new understanding (3) management strategy proposed, including equitable access to any shared resources (4) feasibility of experimental plans, statistics, methodology and design, including provision of sample size calculations, strategies to avoid bias, and preliminary data where appropriate (5) how well risks have been identified, and will be mitigated.

This application proposes to adapt and implement the DIALOG+ for addressing adolescent anxiety and depression in two high violence and high need regions in Columbia. In addition to the intervention efficacy study aim, this application also considers implementation aims (by building capacity for targeted young people, policy stakeholders and researchers, and studying uptake/upscale implementation strategies). Successful completion of the four aims would lead to an evidence-based intervention for adolescents in Columbia, stronger clinical and research capacity for child mental health, strengthening policy-academic-clinical-community partnerships.

There are several methodological and design issues that poses feasibility concerns:
1. Impact evidence for the DIALOG+ on mental health and in adolescents has not been demonstrated (in the proposal): DIALOG+ only showed effectiveness outcome in improving quality of life, but not on mental health outcome measures (e.g., reduction in anxiety and depression). Without these efficacy/effectiveness evidence, it reduces the potential impact of this study.
2. The proposal did not include parents/family members, which may not in line with culture and developmental theory. The family is a big part of adolescent development and support. Parents are also the key sources for crisis and mental illness

management and support for children. However, the proposal study did not mention anything about the role of families/parents in the intervention. Also, parents were not included as stakeholders in Aim 1. It will be critical to include an adolescent mental health expert in the project to oversee the developmentally related design issues.

3. Several aspects of intervention implementation information are missing: For example, who will be introducing DIALOG-A to adolescents in the low mental health professional resources context in Columbia when the number of psychiatrists, psychologists, and psychiatric nurses are limited? What context/care settings will the DIALOG-A be implemented in Columbia when psychiatric facilities are limited (whether both school and primary health center will be included)? What is the estimated intervention dose (amount of time needed for patient-facilitator interaction)? The implementation process/service-workflow is also not well described (e.g., how the intervention is integrated into practice/structure; how the structure and workflow challenges will be addressed).

4. Several aspects of research method/design are missing: (i) the cluster RCT design will be applied (unit of randomization will be clinician), but the description of the design was based on non-cluster design (e.g., the study describe 90 adolescents will be recruited and randomized, but number of clinician and randomization procedure by site was not clearly described); (ii) cluster effect ICC was reported, but the analysis did not adjust for these nesting effect. (iii) Description of the Control condition is not clear. What is the standard care for control condition is not clearly described. (iv)The DIALOG+ intervention appears to be more solution focus, and less psychotherapist/trauma intervention focus. If the intervention is not sufficient for sever anxiety and depression cases, what would be the crisis intervention steps?

5. The potential technology-use and literacy barriers have not been considered. In LMICS, technology (internet, smartphone, tablet) access and use can be limited. A large proportion of targeted age (13-16) might also have low literacy. These technology and education literacy issues have not been considered, which lead to my concern about the feasibility.

6. Multiple communication channels were considered, but feasibility of these approaches in targeted community contexts in Columbia is questionable (what's the feasibility evidence for using Twitter and specific social media channels in adolescents in Columbia; what's the internet availability in the 2 study sites)

Research Environment and People

Please comment on the suitability of the investigator group and the environment where the proposed research will take place, including (1) track record(s) of the individuals in their field(s) and whether they are best-placed to deliver the proposed research (2) level of commitment of host research organisation to supporting the proposed research (3) whether appropriate facilities will be available to the researchers

The research team is well qualified for the proposed project. The team has diverse expertise and successful track records in global health research. The team is likely to successfully develop and implement through a network of partners. The environment to support this research is also strong, with rich resources from related research and clinical networks.

Impact

Please comment on the potential economic and societal impact of the proposed research, including (1) identification of realistic potential improvements to human or population health (2) contribution to relieving disease/disability burden and/or improving quality of life (3) identification of potential impacts of research and plans to deliver these (in the Pathways to Impact statement)

The research Aim and plans are well organized and systematic. Successful completion of the four aims would lead to an evidence-based intervention for adolescents in Columbia, strengthening clinical and research capacity for child mental health, strengthening policy-academic-clinical-community partnerships. It has the potential to improve human or population health through building foundation structures for clinical service improvement and more child mental health intervention service research.

Scientific contribution to adolescent anxiety and depression intervention might be limited, given the scale of trial is small

(with 90 cases), and the intervention (DIALOG-A) has not shown efficacy/effectiveness evidence for improving mental health outcomes (only evidence on improving quality of life, no evidence for improving adolescent mental health). The DIALOG-A intervention also did not include families/parents as sources of support, which is critical in adolescent development and mental health intervention. The intervention also did not integrate effective strategies from the literature for treaing childhood anxiety and depression symptoms. Therefore, the impact of the intervention on sever cases might not be clear.

Ethics

Please comment on any ethical and/or research governance issues, including (1) whether proposed research is ethically acceptable (2) any ethical issues that need separate consideration (3) appropriateness of ethical review and research governance arrangements (4) any potential adverse consequences for humans, animals or the environment and whether these risks have been addressed satisfactorily in the proposal

Given the targeted population will be high-risk and high needs, it is unclear what additional steps will be taken for parents and children with severe anxiety and depression problems/needs if additional service or emergency crisis management is needed. In the high violence context, it is also unclear how children and caregiver safety issues will be addressed if implementers identify potential abuse/violence cases.

Data Management Plan

Please assess whether the data management plan indicates whether the applicants have (or are likely to have) a sound plan for managing the research data funded through the award, taking into account (1) the types, scale and complexity of data being (or to be) managed; (2) the likely long-term value for further research including by sharing data; and (3) the anticipated information security and ethics requirements.

Acceptable.

Resources Requested

Please comment on (1) whether funds requested are essential and justified by the importance and scientific potential of the research (2) investigator time and proposed involvement related to management of the research (3) whether the proposal demonstrates value for money in terms of the resources requested (4) whether any animal use is fully justified in terms of need, species, number, conformance to guidelines

The funding and resource requests are reasonable.

Overall Assessment

Score 1-6

1 - Poor	2 - Good	3 - High	4 - Very High	✔ 5 - Excellent	6 - Exceptional
