# Supplementary material for: Effectiveness of a Patient-Centered Assessment With a Solution-Focused Approach (DIALOG-A) in the Routine Care of Colombian Adolescents With Depression and Anxiety: Protocol for a Multicenter Cluster Randomized Controlled Trial
Source: JMIR Res Protoc. 2023 Feb 8;12:e43401. doi: 10.2196/43401 (PMC9947748; doi:10.2196/43401)
Supplement: Multimedia Appendix 2 [file resprot_v12i1e43401_app2.pdf]

Medical Research Council
2nd Floor David Phillips Building, Polaris House, North Star Avenue, Swindon,
United Kingdom SN2 1ET
Telephone +44 (0) 1793 416200
Web http://www.mrc.ac.uk/

COMPLIANCE WITH THE DATA PROTECTION ACT 1998

In accordance with the Data Protection Act 1998, the personal data provided on this form will be processed by MRC, and may be held on computerised database and/or manual files. Further details may be found in the **guidance notes**

Research Grant Peer Review

MRC Reference: MR/S023674/1

Document Status: With Council

MRC/DFID/NIHR Adolescent Health 2018

Applicant Details

Applicant	Dr Victoria Jane Bird	Organisation	Queen Mary University of London

Title of Research Project

Building resilience in adolescence - improving quality of life for adolescents with mental health problems in Colombia (BRiCs study)

Review Information

Response Due Date	20/12/2018	Reviewer Reference:	011688878

Research Quality

Research Quality: Please comment on the importance and competitiveness of the proposed research, including:

(1) strength of medical or scientific case (2) level of innovation, and whether this is likely to lead to significant new understanding (3) management strategy proposed, including equitable access to any shared resources (4) feasibility of experimental plans, statistics, methodology and design, including provision of sample size calculations, strategies to avoid bias, and preliminary data where appropriate (5) how well risks have been identified, and will be mitigated.

The proposal concerns the development and preliminary evaluation of an adapted psychosocial intervention (DIALOG-A) targeting anxiety and depression in urban and rural Colombian youth. Groundwork for dissemination and implementation of the intervention is included, as well as local public engagement and capacity building initiatives.
1) Strength of case: The adversity faced by adolescents in Colombia is clearly stated. Figures are given for the prevalence of anxiety and depression, numbers accessing any treatment, and the proportion of treatment offers that reach a minimum standard. It would be helpful to compare these figures to those for a socially settled, non-LMIC context (particularly given recent UK figures highlighting the difficulties young people face in accessing mental health care); and to specify the constituents of minimally adequate care, and care falling below this standard. The proposal states that there are too few trained professionals in Colombia to train to deliver the evidence-based interventions recommended in HICs, so a more readily deliverable approach is required: there is good evidence that unadapted DIALOG can be delivered, with good outcomes, by a range of workers to adults with psychosis, across international settings. Outcomes for anxiety and depression are not given and DIALOG has not yet been offered to young people. Any indication that the intervention could be helpfully adapted to suit this anxious/depressed exclusively mid-adolescent population is missing from the proposal - both in terms of practical adaptability and proposed mechanisms of change - I understand DIALOG to be intended to create

more equitable, personalised, empowering and solution-focused conversations between health professionals and patients - particularly relevant in adult psychosis services, where patients lack insight, often engage reluctantly with care, may dislike taking medication (which remains the first line treatment), may be particularly sensitive to power dynamics in relationships, and have often received interventions against their will. It is not clear how the same intervention would be expected to work for helpseeking adolescents presenting with conditions that are usually treated psychosocially.
2) Level of innovation: The study would potentially provide new information about the self- and service-identified needs of anxious and depressed youth in Colombia, and facilitators of, and barriers to, the delivery of interventions and conduct of research - but from qualitative analyses of relatively small numbers of participants (compared to the target populations). It could also lead to the development of a new intervention for this setting.
3) Management strategy & sharing resources: The team is very experienced and this aspect of the proposal looks strong to me.
4) Experimental plans: I have some reservations about this. There are no preliminary data on the feasibility of the recruitment plan (though I appreciate this is a feasibility study, failure to recruit is still a risk, and a preliminary survey of willingness to engage, or comparison to a similar study would be useful). Consent procedures need to be refined - what is the legal age at which a child can provide sole consent, and if needed, how would parents be involved, and how will confidentiality issues be managed. As a feasibility study, the criteria for judging the study to be feasible should be stated a priori. All measures need validation data for the target population.
5) Risks & mitigation: the feasibility study (WP2) is only relevant if DIALOG can successfully be adapted in WP1, and the implementation work in WP3 is only relevant if DIALOG proves feasible - the possibilities of failure to create an appropriate adaptation, and evidence of infeasibility need consideration. Information on establishing protocols to ensure clinical safety and to record adverse events is needed.

Research Environment and People

Please comment on the suitability of the investigator group and the environment where the proposed research will take place, including (1) track record(s) of the individuals in their field(s) and whether they are best-placed to deliver the proposed research (2) level of commitment of host research organisation to supporting the proposed research (3) whether appropriate facilities will be available to the researchers

1) The team is highly experienced and well-placed to deliver the study. Paediatric expertise is provided only in Colombia - there could be value in having a UK-based collaborator with expertise in treating adolescent anxiety and depression. A co-applicant with statistical expertise would also strengthen the proposal.
2) Host commitment - this looks strong
3) Facilities - these appear good in both settings.

Impact

Please comment on the potential economic and societal impact of the proposed research, including (1) identification of realistic potential improvements to human or population health (2) contribution to relieving disease/disability burden and/or improving quality of life (3) identification of potential impacts of research and plans to deliver these (in the Pathways to Impact statement)

Should the study be successful, the impact would be significant. More could be made of the impact of findings contrary to expectation and how the team would make use of the data collected and progress in these circumstances.

Ethics

Please comment on any ethical and/or research governance issues, including (1) whether proposed research is ethically acceptable (2) any ethical issues that need separate consideration (3) appropriateness of ethical review and research governance arrangements (4) any potential adverse consequences for humans, animals or the environment and whether these risks have been addressed satisfactorily in the proposal

These have been reasonably well-considered. There could be more consideration of: consent procedures, parental involvement and confidentiality, patient safety and the possibility of adverse events. Plans for formal ethical review are appropriate.

Data Management Plan

Please assess whether the data management plan indicates whether the applicants have (or are likely to have) a sound plan for managing the research data funded through the award, taking into account (1) the types, scale and complexity of data being (or to be) managed; (2) the likely long-term value for further research including by sharing data; and (3) the anticipated information security and ethics requirements.

This looks well-considered, for all types of data collected.

Resources Requested

Please comment on (1) whether funds requested are essential and justified by the importance and scientific potential of the research (2) investigator time and proposed involvement related to management of the research (3) whether the proposal demonstrates value for money in terms of the resources requested (4) whether any animal use is fully justified in terms of need, species, number, conformance to guidelines

The costs are carefully detailed. The PPI component carries a relatively high cost for the scientific return, but may be a key component of engaging the target group and promoting the study.

Overall Assessment

Score 1-6

1 - Poor	✔ 2 - Good	3 - High	4 - Very High	5 - Excellent	6 - Exceptional
