# Supplementary material for: Effectiveness of a Patient-Centered Assessment With a Solution-Focused Approach (DIALOG-A) in the Routine Care of Colombian Adolescents With Depression and Anxiety: Protocol for a Multicenter Cluster Randomized Controlled Trial
Source: JMIR Res Protoc. 2023 Feb 8;12:e43401. doi: 10.2196/43401 (PMC9947748; doi:10.2196/43401)
Supplement: Multimedia Appendix 3 [file resprot_v12i1e43401_app3.pdf]

**Medical Research Council**
2nd Floor David Phillips Building, Polaris House, North Star Avenue, Swindon,
United Kingdom SN2 1ET
**Telephone +44 (0) 1793 416200**
**Web http://www.mrc.ac.uk/**

**COMPLIANCE WITH THE DATA PROTECTION ACT 1998**

In accordance with the Data Protection Act 1998, the personal data provided on this form will be processed by MRC, and may be held on computerised database and/or manual files. Further details may be found in the **guidance notes**

# Research Grant Peer Review

MRC Reference: MR/S023674/1

Document Status: With Council

## MRC/DFID/NIHR Adolescent Health 2018

**Applicant Details**

| | | | |
|---|---|---|---|
| Applicant | Dr Victoria Jane Bird | Organisation | Queen Mary University of London |

**Title of Research Project**

Building resilience in adolescence - improving quality of life for adolescents with mental health problems in Colombia (BRiCs study)

**Review Information**

| | | | |
|---|---|---|---|
| Response Due Date | 30/11/2018 | Reviewer Reference: | 144701187 |

**Research Quality**

Research Quality: Please comment on the importance and competitiveness of the proposed research, including:

*(1) strength of medical or scientific case (2) level of innovation, and whether this is likely to lead to significant new understanding (3) management strategy proposed, including equitable access to any shared resources (4) feasibility of experimental plans, statistics, methodology and design, including provision of sample size calculations, strategies to avoid bias, and preliminary data where appropriate (5) how well risks have been identified, and will be mitigated.*

This proposal is important as it addresses gaps in the knowledge of effective treatments for youth with anxiety and depression in low-middle income countries. It is innovative as it uses technology as a cost-effective tool, and findings could increase our understanding about the most effective ways to bridge the mental health treatment gap. The research strategy is convincing and coherent, with good quality in the methodology. However, one of the things I missed to see was a specific measure that evaluates resilience, given that is one of the purpose of the project. I strongly suggest to include this. Also, the intervention DIALOG+ has not been fully described, nor the specific cultural, language, and developmental adaptations that they proposed to make to repurpose the intervention to DIALOG-A. The intervention is at the core of the project, and one of the main objectives is to develop a culturally adapted intervention, but the process to achieve this deserves more attention. For example, there are WHO guidelines for culturally adapting instruments that propose a six step process that includes several back-to-back translations, the review of the translation by an expert panel, and the piloting. A more detailed explanation of this should be included in the proposal. In the same way, I missed to see literature regarding the effectiveness of using technology in mental health, as this is the delivery mode of the proposed intervention. One of the strengths of the proposal is the sharing of the knowledge and resources with the community, but remains unclear how this will be done, particularly the part where they talked about the project being open to the public and the intervention app

available without training, this part needs more clarification. Involving multiple stakeholders in the process of adapting and implementing a new intervention is crucial and has been identified by the research team. However, a more concrete plan needs to be established on how to do so. The part of involving the adolescents in the video film project is described through the document, but not the specific actions they will take to include multiple stakeholders at all stages of the research project, including the selection procedure of the members.

### Research Environment and People

*Please comment on the suitability of the investigator group and the environment where the proposed research will take place, including (1) track record(s) of the individuals in their field(s) and whether they are best-placed to deliver the proposed research (2) level of commitment of host research organisation to supporting the proposed research (3) whether appropriate facilities will be available to the researchers*

A main strength of this project is the talent and expertise of the team. Their records showed successful academic careers for the team members, a strong commitment of both sites (UK and Colombia) to the project, and the support of their respective institutions. There is a balance between senior researchers and mid-career researchers. A high level of expertise on international mental health and grant writing is present. The Colombian team will have the appropriate facilities required to implement the research project, as well as the UK team. However, I missed to see in their publication lists more of their work on the area of anxiety, and adolescent mental health. The publication lists of most of them illustrates a stronger expertise on depression and adults. As these are short bios and selected publications, I will suggest to include more information related to the specific target of the research project: anxiety and depression in adolescents.

### Impact

*Please comment on the potential economic and societal impact of the proposed research, including (1) identification of realistic potential improvements to human or population health (2) contribution to relieving disease/disability burden and/or improving quality of life (3) identification of potential impacts of research and plans to deliver these (in the Pathways to Impact statement)*

The project has the potential impact to improve mental health and contribute to the quality of life of adolescents in Colombia. The situation of the country places this population as a vulnerable one, and in great need of mental health services, thus this project is highly relevant. This project has the potential to be further use to answer additional research questions. Particularly as research in low-middle income countries and capacity building is scare, part of the data could be further analyzed to document providers' perspectives on the train-the-trainer model, use of technology, among others.

### Ethics

*Please comment on any ethical and/or research governance issues, including (1) whether proposed research is ethically acceptable (2) any ethical issues that need separate consideration (3) appropriateness of ethical review and research governance arrangements (4) any potential adverse consequences for humans, animals or the environment and whether these risks have been addressed satisfactorily in the proposal*

The records of the team members of this research proposal reflect an extensive expertise on conducting research on the area of mental health. The research proposal stated that the research protocol will be sent for approval to the IRB of the university from Colombia, but did not mention the UK IRB. I will suggest to also submit the research protocol for review to the IRB of the UK institution.

### Data Management Plan

*Please assess whether the data management plan indicates whether the applicants have (or are likely to have) a sound plan for managing the research data funded through the award, taking into account (1) the types, scale and complexity of data being (or to be) managed; (2) the likely long-term value for further research including by sharing data; and (3) the anticipated information security and ethics requirements.*

The data management plan shows a sound plan for managing research data. As a note, the first link on page 3, under section 7 does not open.

**Resources Requested**

*Please comment on (1) whether funds requested are essential and justified by the importance and scientific potential of the research (2) investigator time and proposed involvement related to management of the research (3) whether the proposal demonstrates value for money in terms of the resources requested (4) whether any animal use is fully justified in terms of need, species, number, conformance to guidelines*

The funds requested seem justified by the potential and scientific potential of the research. A particular strength of this project is the building capacity part, in where UK researchers will mentor Colombian researchers and will share knowledge about leadership skills, research methods, and finance. Thus, expanding the impact of their learning to other potential research projects that are carried out in the future. I suggest that the building capacity part be described in more detailed by explaining how scale-up will take place and the specific agenda to expand this knowledge to the community (i.e., other universities/organizations/public institutions in Colombia). How will this be done? Through conferences, webinars, exchange of faculty, and/or during training programs? The time commitment to the work and value for money are appropriate. My only suggestion will document better the film initiative, to consider cultural issues. Also, it will be worth to consider other activities that may have a bigger impact? Such as providing adolescents with opportunities to engage in social groups activities like sports and arts (the proposal mentioned that about 50% of the adolescents in Colombia do not have this opportunity).

**Overall Assessment**

*Score 1-6*

| | | | | | |
|---|---|---|---|---|---|
| 1 - Poor | 2 - Good | 3 - High | 4 - Very High | ✔ 5 - Excellent | 6 - Exceptional |
